# Treating latent TB in primary care: a survey of enablers and barriers among UK General Practitioners

**DOI:** 10.1186/s12879-015-1091-9

**Published:** 2015-08-13

**Authors:** Christina Atchison, Dominik Zenner, Lily Barnett, Manish Pareek

**Affiliations:** Department of Primary Care and Public Health, Imperial College London, Room 332, Reynolds Building, Charing Cross Campus, London, W6 8RF UK; Centre for Infectious Disease Surveillance and Control, Public Health England, London, NW9 5EQ UK; Centre for Infectious Disease Epidemiology, University College London, London, NW1 2DA UK; National Institute for Health Research Health Protection Research Unit in Respiratory Infections, Imperial College London, London, W6 8RF UK; Department of Infection, Immunity and Inflammation, University of Leicester, Leicester, LE1 9HN UK

## Abstract

**Background:**

Treating latent tuberculosis infection (LTBI) is an important public health intervention. In the UK, LTBI treatment is delivered in secondary care. Treating LTBI in the community would move care closer to home and could increase uptake and treatment completion rates. However, healthcare providers’ views about the feasibility of this in the UK are unknown. This is the first study to investigate perceived barriers and enablers to primary care-based LTBI treatment among UK general practitioners (GPs).

**Methods:**

A national survey amongst 140 randomly sampled UK GPs practising in areas of high TB incidence was performed. GPs’ experience and perceived confidence, barriers and enablers of primary care-based LTBI treatment were explored and multivariable logistic regression was used to determine whether these were associated with a GP’s willingness to deliver LTBI treatment.

**Results:**

One hundred and twelve (80 %) GPs responded. Ninety-three (83 %; 95 % CI 75 %–89 %) GPs said they would be willing to deliver LTBI treatment in primary care, if key perceived barriers were addressed during service development. The major perceived barriers to delivering primary care-based LTBI treatment were insufficient experience among GPs of screening and treating LTBI, lack of timely specialist support and lack of allied healthcare staff. In addition, GPs felt that appropriate resourcing was key to the successful and sustainable delivery of the service. GPs who reported previous experience of screening or treatment of patients with active or latent TB were almost ten times more likely to be willing to deliver LTBI treatment in primary care compared to GPs with no experience (OR: 9.98; 95 % CI 1.22–81.51).

**Conclusions:**

UK GPs support primary care-based LTBI treatment, provided they are given appropriate training, specialist support, staffing and financing.

**Electronic supplementary material:**

The online version of this article (doi:10.1186/s12879-015-1091-9) contains supplementary material, which is available to authorized users.

## Background

The World Health Organisation (WHO) recommended that diagnosis and treatment of latent tuberculosis infection (LTBI) could play a key role in TB elimination [1, 2], and the new WHO post-2015 global TB strategy has placed more importance on tackling LTBI, especially in low incidence countries [2].

In the United Kingdom (UK) the majority of TB cases occur amongst individuals who were born in high incidence countries outside the UK, such as the Indian subcontinent and sub-Saharan Africa [3]. Approximately 85 % of TB cases amongst non-UK born individuals are diagnosed more than two years after entering the UK [4]. Molecular epidemiological studies indicate that the majority of these cases occur as a result of LTBI reactivation, often acquired overseas [5]. Therefore, whilst it remains important to prevent new TB transmissions, addressing tuberculosis in migrants including LTBI in selected migrants to the UK needs to be tackled if an epidemiological impact on TB incidence is to be made.

In the UK screening and treatment for LTBI has been recommended by the National Institute of Clinical Excellence (NICE) since 2006 [6], but implementation is variable [7]. Recent studies have found that targeted LTBI screening and treatment of migrants from high incidence countries is feasible, and cost effective [8–12]. Following successful pilot studies across the UK [11–13], primary care-based migrant LTBI screening has become a key intervention in the collaborative TB strategy for England 2015–2020 [14]. However, to date, there has been little focus on how best to clinically manage migrants with LTBI. Currently in the UK, LTBI treatment is delivered in secondary care, but uptake and adherence to treatment among migrants is often poor [15]. A recent study showed that providing specialist clinics in primary care settings led to improved access, lower travel costs and shorter waiting times for patients [16]. Thus, LTBI treatment in the community and providing care closer to home, could be more acceptable and make treatment more accessible to patients. This in turn could result in increased uptake and treatment completion.

There is scarce evidence on the feasibility and acceptability of LTBI treatment in primary care. We hypothesise that there are perceived and real barriers in the UK to delivering LTBI treatment outside secondary care. We aim to explore the perceived barriers and enablers to primary care-based LTBI treatment among UK general practitioners (GPs). We also explore GPs’ views with regards to the type of delivery model and support package that would be required to provide a coordinated primary care-based LTBI service for migrant communities in the UK. The information collected in this study is important in order to inform policy and the direction of further research into primary care-based LTBI treatment.

## Methods

### Study design and sampling of study population

A mixed method online questionnaire-based study was conducted between February and April 2014 among UK general practitioners.

The sampling frame was a register of all General Practices in high TB burden local authority areas in England (incidence ≥40 TB cases per 100,000 population) according to the three-year average reported incidence in 2010–2012 [3]. Multi staged random sampling was utilised. We identified 14 high incidence local authority areas and these are broadly consistent with Clinical Commissioning Groups (CCGs) health authorities that include all General Practices in their geographical area. We randomly sampled half of these CCGs then randomly selected 20 General Practices from each CGG. Within each practice, a single GP partner or salaried GP was randomly selected to participate in the survey. A total of 140 GPs were invited to complete the online questionnaire.

Sample size calculations were based on the main outcome the percentage of GPs willing to deliver a primary care-based LTBI treatment model. We estimated this to be 60 %, based on studies exploring GPs’ attitudes to shifting drug misuse services into primary care in the UK [17, 18] as there were no studies in the literature exploring GPs’ attitudes to LTBI treatment in primary care. A sample size of 140 GPs was required to give a +/- 8 % level of precision around our estimate at the 95 % confidence level.

### Ethical approval

Ethical approval was sought from the Imperial College London, NHS England and Public Health England Research Ethics Committees who advised that, in accordance with their research ethics specifications and guidance, it was not required for this study because it was performed under the remit of a service evaluation of National Health Service (NHS) staff.

### Questionnaire development and design

A 16-point confidential electronic questionnaire (see Additional file 1) was developed and administered online using SelectSurvey®. The questionnaire was designed in consultation with GPs with a specialist interest in TB, TB physicians and public health specialists to improve content validity, and a pilot study was conducted amongst eight GPs to improve face validity. From the pilot study we determined that the questionnaire took 5 to 10 minutes to complete.

The questionnaire sought to identify key enablers and barriers of LTBI treatment in primary care and GPs’ willingness to deliver a primary care-based LTBI treatment model. The questionnaire also included questions about the GP’s profile: qualifications, duration of GP experience, number of GPs based in the general practice, and type of area served by the general practice (e.g. urban, suburban, rural). Other questions included: whether screening and treating LTBI in migrants was perceived to be an important strategy for reducing TB incidence in the UK, whether TB was perceived as a health problem in the GP’s practice population, the current practice of the GP and previous experience of screening and treating active and latent TB, and a GP’s perceived level of confidence in screening and treating LTBI. Most questions in the final questionnaire had a closed yes/no or multiple choice format with an open answer option. A five-point Likert-type scale from *strongly agree* to *strongly disagree* was used for all questions that required assessment of the strength of opinion [19].

A cover letter and link to the online questionnaire was emailed to GPs with two reminder emails sent four and six weeks after the initial mailing respectively. This was followed up with a telephone call if needed. GPs were provided with a cover letter and a brief statement to read on the first page of the online self-administered questionnaire explaining the purpose of the study. Consent was implied by the receipt of a completed questionnaire.

### Statistical analysis

Descriptive statistics (frequencies and percentages) were calculated for questionnaire items where appropriate.

For qualitative analysis of textual responses to open-ended questions open coding was performed independently by two of the authors (CA and LB). Discussions took place to develop a coding frame based on themes emerging from the open codes, using a process of progressive focusing [20]. This coding frame included both descriptive and conceptual categories [21]. All textual responses were then systematically coded. During this stage, further discussions took place in order to further adapt and refine the coding frame. This process aimed to ensure that the emerging themes identified were grounded in the data collected [21], but also relevant to the study aims.

Univariable and multivariable logistic regression models to calculate odds ratios (ORs) with 95 % confidence intervals (CIs) were developed to determine whether a priori hypothesised factors and barriers identified in the qualitative analysis were associated with a GP’s willingness to deliver a primary care-based LTBI treatment model. Independent variables tested included: (1) years of GP experience (2) whether TB was perceived as a health problem in the GP’s practice population, (3) a GP’s previous experience of screening or treatment of patients with active or latent TB, (4) a GP’s perceived level of confidence in a number of specific aspects of screening and treating LTBI, and (5) a number of barriers to LTBI treatment in primary care identified by the GPs. Variables that yielded a likelihood ratio (LR) test result of *p* < 0.05 in univariable analyses were included in the multivariable analysis [22]. We assessed for collinearity (using variance inflation factor) and interaction between independent variables where appropriate.

STATA/SE Version 13 software (STATA College Station, Texas, USA) was used for all statistical analyses.

## Results

### Response rate

Of the 140 GPs invited to complete the survey 112 (80 %) responded. Those who did not respond were invited to give a reason during a follow-up phone call. All those who did not participate (*n* = 28) stated they declined due to lack of time.

### Profile of GP respondents

Table 1 shows the profile of the 112 GPs who took part in the study. Eighty-six (77 %; 95 % CI 68 %–84 %) worked in a general practice serving an urban population. The median number of years of GP experience was 11.5 years (range: 2 to 40 years). Twenty-four (21 %; 95 % CI 15 %–30 %) GPs had specialist interest accreditation most commonly for Diabetes (*n* = 6) and Substance Misuse (*n* = 7). One GP had specialist interest accreditation in Respiratory Medicine. Limited information was also collected on the 28 GPs who did not complete the survey (Table 1). There was no significant difference between responders and non-responders in terms of type of area served by the general practice (*p* = 0.18) and number of GPs based in the general practice (*p* = 0.96).

**Table 1:** Profile of GP responders (*n* = 112) and non-responders (*n* = 28)

Characteristic	Responders No. (%; 95 % CI)	Non-responders No. (%; 95 % CI)	*p*-value^a^
Type of area served by the general practice:			
Urban	86 (76.7; 67.9–83.8)	17 (60.8; 40.8–77.6)	
Suburban	20 (17.9; 11.7–26.2)	7 (25.0; 11.8–45.3)	
Rural	3 (2.7; 0.9–8.1)	2 (7.1; 1.6–26.3)	
Mixed urban/rural	3 (2.7; 0.9–8.1)	2 (7.1; 1.6–26.3)	0.18
Number of GPs based in the general practice:			
1–5	56 (50.0; 40.7–59.3)	13 (46.4; 28.2–65.7)	
6–10	41 (36.6; 28.1–46.0)	12 (42.9; 25.2–62.5)	
11–15	10 (8.9; 4.8–15.9)	2 (7.1; 1.6–26.3)	
>15	5 (4.5; 1.8–10.4)	1 (3.6; 0.4–23.7)	0.96
Years of GP experience:			
1–5	18 (16.1; 10.3–24.2)	-	
6–15	52 (46.4; 37.3–55.8)	-	
16–30	35 (31.3; 23.3–40.5)	-	
>30	7 (6.2; 3.0–12.7)	-	-
Country of primary medical qualification:			
UK	79 (70.5; 61.3–78.3)	-	
Other European countries	10 (8.9; 4.8–15.9)	-	
Asian Subcontinent countries	20 (17.9; 11.7–26.2)	-	
Sub-Saharan Africa countries	2 (1.8; 0.4–7.0)	-	
Australia	1 (0.9; 0.1–6.2)	-	-
GP with special interest accreditation:			
Yes	24 (21.4; 14.7–30.1)	-	-

^a^Fisher’s exact test

### Perceptions, experience and current practice

Of the GPs responding 57 (51 %; 95 % CI 42 %–60 %) perceived TB as a health problem in their general practice population. Seventy-six (68 %; 95 % CI 59 %–76 %) GPs either strongly agreed or agreed that LTBI screening and treatment of migrants from high incidence countries was an important strategy for reducing TB incidence in the UK, and only four GPs (4 %; 95 % CI 1 %–9 %) disagreed with this statement.

Ninety-seven (87 %; 95 % CI 79 %–92 %) GPs reported that they had not screened for or treated LTBI as part of their practice as a GP of whom sixty-two (64 %; 95 % CI 54 %–73 %) GPs stated that they would refer individuals for LTBI screening and treatment to secondary care. Thirty-three (29 %; 95 % CI 22 %–39 %) of all GPs who responded to the survey reported previous experience of screening or treatment of patients with active or latent TB; this was predominantly in the context of hospital infectious diseases (*n* = 7) or respiratory medicine (*n* = 14) placements as junior doctors, or as part of an overseas post (*n* = 5).

**Fig. 1:**
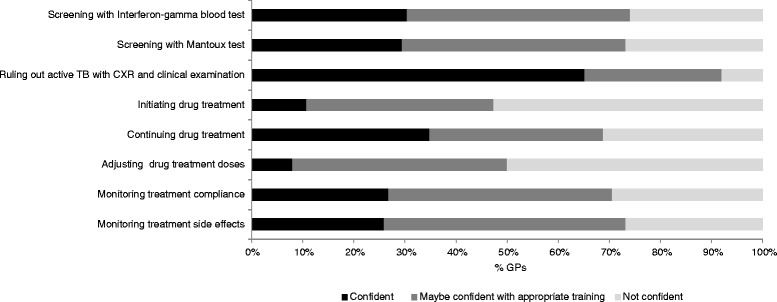
GPs’ perceived level of confidence in screening and treating LTBI, *n* = 112. *Abbreviation: CXR = chest x-ray

Seventy-three (65 %; 95 % CI 56 %–74 %) GPs reported they felt confident in ruling out active TB with chest x-ray and clinical examination. Only 12 (11 %; 95 % CI 6–18) GPs reported they felt confident in initiating LTBI drug treatment and nine (8 %; 95 % CI 4–15) GPs reported confidence in adjusting drug doses of individuals on LTBI treatment (Fig. 1).

### Perceived barriers and enablers for primary care-based LTBI treatment

Fifty-nine (53 %; 95 % CI 43 %–62 %) GPs either strongly agreed or agreed that there was a need for a primary care-based GP-led service for LTBI treatment for adult migrants, 21 (19 %; 95 % CI 12 %–27 %) GPs disagreed with this statement. Six (5 %; 95 % CI 2 %–12 %) GPs stated that they would be willing to deliver LTBI treatment in primary care and a further 87 (78 %; 95 % CI 69 %–85 %) GPs said they would be willing to deliver LTBI treatment in primary care if key barriers were addressed and suggested enablers considered when developing the service model.

Four main themes emerged from GPs regarding perceived barriers and enablers for delivering LTBI treatment in primary care:

#### Training

The major perceived barrier was insufficient experience among GPs regarding all aspects of LTBI screening and treatment (82 %; 95 % CI 74 %–88 %). Eighty-eight (79 %; 95 % CI 70 %–85 %) GPs suggested that specific training in LTBI screening and treatment would be an important enabler. Interactive workshops or structured online learning tools were specified as the preferred methods of training. Other suggestions included a handbook, case-based discussions and short placements within hospital-based TB specialist teams.

#### Timely access to specialist support

Lack of timely support from specialist TB services was also a major concern (61 %; 95 % CI 51 %–69 %). Sixty-three (56 %; 95 % CI 47 %–65 %) GPs stated that easy access and timely support from specialist TB teams would facilitate delivering LTBI treatment in primary care as well as developing clear referral pathways to secondary care for more complex cases.

#### Resources

Seventy-six (68 %; 95 % CI 59 %–76 %) GPs stated that more resources would be required. Suggestions included more GP appointments, longer GP appointments, appointing a GP with TB or respiratory medicine special interest accreditation, appointing a community-based TB nurse, and dedicated phlebotomist and community pharmacist sessions.

#### Financial incentives and service contracts

Specific funding was viewed as an important enabler (63 %; 95 % CI 53 %–71 %). Many GPs did not know or felt unable to comment on the type of funding or financial resources required but the majority indicated that a feasible and acceptable level of funding was key to the successful and sustainable delivery of the service. Some GPs recommended “*adequate to cover hourly GP rate*” and others mentioned “*GP with special interest accreditation level remuneration*”. Several GPs suggested putting contractual arrangements in place such as a Locally Enhanced Service (LES) or Quality and Outcomes Framework (QOF) targets.

### Multivariable analysis

For the purpose of the analysis a GP’s willingness to deliver LTBI treatment in primary care was recoded into a binary outcome: “Yes” (willing to deliver LTBI treatment in primary care/willing to deliver LTBI treatment in primary care if key barriers and enablers taken into account) and “No” (not willing to deliver LTBI treatment in primary care under any circumstances). No individual barrier or perceived level of confidence in any particular aspect of screening and treating LTBI was associated with a GP’s willingness to deliver LTBI treatment in primary care. In the final multivariable model previous experience of screening or treatment of patients with active or latent TB was the only factor associated with a GP’s willingness to deliver LTBI treatment in primary care. GPs who reported previous experience of screening or treating active or latent TB were almost ten times more likely to be willing to deliver LTBI treatment in primary care compared to GPs with no experience (OR: 9.98; 95 % CI 1.22–81.51) (Table 2).

**Table 2:** Multivariable model of factors affecting a GP’s willingness to deliver LTBI treatment in primary care^a^

Factor	OR (95 % CI)	*p*-value
^b^Years of GP experience	1.01 (0.95–1.07)	0.81
^c^TB perceived as a problem in the GP’s practice population	1.62 (0.43–6.14)	0.48
^c^Previous experience of screening or treating active or latent TB	9.98 (1.22–81.51)	0.03

^a^only factors that yielded *p* < 0.05 in univariable analysis were included in the multivariable model

^b^continuous numerical variable; ^c^binary variable (Yes/No)

## Discussion

There is a high level of support among UK GPs for primary care-based treatment of LTBI. This was an unexpected finding as moving the service into primary care would involve additional responsibilities for General Practices. There were a number of key findings. GPs clearly identified some major barriers to delivering LTBI treatment in primary care including perceived lack of experience in LTBI, lack of timely access to specialist support and resource limitations. These barriers need addressing if the GP support for moving LTBI treatment into primary care is to be sustained. However, the results are very encouraging and provide a strong platform to support the development and trialling of models of primary care-based LTBI treatment in the UK. In light of our findings we propose a primary care-based LTBI model delivered by GPs with special interest in TB, who in addition to their core professional roles would provide specialist LTBI screening and treatment services supported by interaction with hospital TB specialists. To deliver the service successfully there will need to be appropriate teaching and training, ready access to secondary care, sufficient allied healthcare staff, in particular pharmacist and phlebotomist, and contractual agreements which include sufficient funding and resources to cope with the additional workload of providing a new service.

### Strengths and limitations

To our knowledge this is the first study to explore barriers and enablers to primary care-based LTBI treatment among GPs. The response rate was high, possibly attributable to efforts made to engage CCGs through face-to-face meetings and information events for GPs to raise the profile of the study. An additional strength was that we had no missing data. This was because the survey software we used allowed us to set questions to “answer to this question is required”. Therefore, a responder could not proceed to subsequent questions until an answer was given. However, our study has some limitations. Response bias cannot be completely ruled out, we might expect that the 28 GPs who did not respond would be less willing to deliver LTBI treatment in primary care as all declined to respond due to lack of time and therefore might be less willing to deliver a service which could increase their workload. We compared several general practice characteristics between responders and non-responders and there was no significant difference. We attempted to minimise measurement bias by involving a panel of TB and public health experts in questionnaire design and by piloting the study first. However, feedback following completion of the main study identified that some questions needed to be clearer, or could have been refined to be made more intuitive and relevant to participants. This feedback highlights the importance of comprehensively piloting a questionnaire prior to carrying out a survey-based study. For example, some questions did not distinguish LTBI screening from treatment, and these are different aspects which could have been explored in more detail if GPs were asked about these aspects separately. However, the wording of the questions regarding barriers and enablers specified LTBI treatment only. Previous studies looking at LTBI screening in primary care did not explore these issues in relation to LTBI treatment which is what makes our study unique. The use of a self-administered questionnaire ensured that data could be reliably aggregated and that the study could be replicated in different settings in the future. However, focus groups or individual interviews may have provided more detailed information. Our findings are based on a random sample of GPs and findings from this study are likely generalisable to UK GPs practising in areas of high TB incidence. It was appropriate to focus on the views of GPs in high TB incidence areas as this would be the setting of any LTBI treatment service in the future.

### Findings related to previous studies

Moving patient care from hospitals into the community has been a UK-wide priority for over a decade [23, 24]. Successfully and safely moving the monitoring and treatment of non-insulin dependent diabetes patients from secondary care to primary care with a new general practice contract and QOF targets in 2003 was a major achievement in the UK [25]. Its implementation was supported by financial rewards and national targets. Many GPs in our study may have drawn from lessons learnt with diabetes when indicating that adequate funding was the key to a successful primary care-based LTBI treatment model. In fact, several GPs suggested putting contractual arrangements in place similar to those currently in place for diabetes management in primary care.

Internationally there has recently been interest in moving drug misuse services from secondary care to primary care [17, 18]. Studies of attitudes and knowledge of GPs, secondary care staff and patients in relation to drug misuse services have shown similar results to our study. GPs increasingly support a role for primary care in delivering the service, although similar to our findings a lack of confidence and experience in managing drug misusers, and lack of resources were identified as major barriers [17, 18]. GPs also viewed financial incentives for delivering the service as important [17, 18]. Interestingly, in these studies some secondary care staff and patients felt that GPs would not be able to deliver the same quality of care because of their limited experience and availability of appointment times [17, 18].

### Implications and future research

Our findings are important in order to inform UK policy and the direction of further research into primary care-based LTBI treatment. Future studies to assess acceptability, adherence and safety of LTBI treatment in primary care should be implemented with careful consideration of how to address perceived barriers. The responses from GPs from our survey could help overcome these barriers. Finally, exploring GPs’ views is only one side of the story. Moving LTBI treatment into primary care will also impact on patients and specialist TB services. Future work is required to explore the attitudes and beliefs of these stakeholders. The information collected could also be of interest to policymakers facing similar decisions overseas.

## Conclusions

UK GPs support primary care-based LTBI treatment, provided they are given appropriate training, specialist support, staffing and financing. Our results provide a reliable contemporary picture of the enablers and barriers to primary care-based LTBI treatment in the UK. As such we provide the first evidence to rethink the models of care we offer to those migrants identified to have LTBI. This study should serve a template for other countries (both in Europe and North America) to evaluate their models of care.

## Additional file

Additional file 1:
**The LTBI GP survey questionnaire.** (PDF 291 kb)

## Abbreviations

LTBI: Latent tuberculosis infection
WHO: World Health Organisation
UK: United Kingdom
GP: General practitioner
NICE: National Institute of Clinical Excellence
NHS: National Health Service
