# Supplementary material for: Treating latent TB in primary care: a survey of enablers and barriers among UK General Practitioners
Source: BMC Infect Dis. 2015 Aug 13;15:331. doi: 10.1186/s12879-015-1091-9 (PMC4535609; doi:10.1186/s12879-015-1091-9)
Supplement: Additional file 1: — The LTBI GP survey questionnaire. (PDF 291 kb) [file 12879_2015_1091_MOESM1_ESM.pdf]

Public Health England

A survey of GPs' attitudes towards primary care-based latent TB management for healthy adult migrants

Latent TB infection (LTBI) means the presence of Mycobacterium tuberculosis bacteria in the body as evidenced by a significant reaction to a Mantoux tuberculin skin test or positive interferon gamma release assay. Unlike active tuberculosis disease, a person with LTBI does not have any symptoms nor is he or she infectious.

In order to better understand current primary care practices with respect to the management of LTBI, and to understand key enablers and barriers for future provision of this service within primary care, we are conducting a survey of GPs working in practices within local authorities with high TB incidence. We would be extremely grateful if you could take the time to complete this survey.

The survey questionnaire will take 5-10 minutes to complete. Many thanks for your participation.

A. Personal Details

1. Which CCG does your practice belong to:

☐Brent ☐Ealing ☐Greenwich ☐Haringey ☐Harrow ☐Hillingdon

☐Hounslow ☐Leicester ☐Luton ☐Newham ☐Redbridge ☐Slough

☐Tower Hamlets ☐Waltham Forest

2. Is your GP practice mostly: ☐Rural ☐Urban ☐Suburban ☐Mixed urban/rural
3. Number of GPs working in your practice: _ _ _
4. Number of years as a GP: _ _ _
5. Country of primary medical qualification: _
6. Are you a GP with Special Interest accreditation: ☐No

   ☐Yes, your special interest: _ _ _ _ _ _ _ _ _ _ _ _ _ _ _ _ _

B. Latent TB: Perception of local burden and current management

7. Latent TB screening and treatment of migrants from high incidence countries is a key strategy to reduce TB disease incidence in the UK (please circle):

   Strongly disagree / Disagree / Impartial / Agree / Strongly agree

8. Do you think TB is a health problem in your practice population: Yes/No/Don't Know
9. Do you screen or treat for latent TB in primary care as part of your current GP practice: Yes/No
10. If you answered No to Qu. 9, do you refer patients with latent TB to specialist services for screening or treatment: Yes/No/ Don't Know

    If Yes, which speciality: ☐Infectious Diseases ☐Respiratory ☐Other, please specify: _

    If Yes, which patient groups: ☐All ☐HIV positive ☐IVDUs ☐Children ☐Migrants ☐Other, please specify: _

11. Do you have experience in the screening or treatment of patients with active or latent TB: Yes/No

    If Yes, at what level: ☐GP ☐Consultant ☐SpR/StR ☐SHO ☐PRHO/FY1/FY2 ☐Other, please specify: _

    If Yes, where did you gain your experience: ☐Respiratory placement in hospital ☐ Infectious Diseases placement in hospital ☐General practice ☐Other, please specify: _

    If Yes, how long has your experience been: ☐<1 month ☐1-6 months ☐6-12 months ☐>12 months ☐Other, please specify: _

12. Which of the following aspects of managing <u>healthy adult migrants</u> with latent TB would you be confident doing:

	Confident	Maybe, with appropriate training	Not confident
Screening for latent TB with Mantoux test			
Screening for latent TB with Interferon-gamma blood test			
Ruling out active TB with chest X-ray and clinical examination			
Initiating drug treatment with isoniazid and rifampicin or isoniazid alone			
Continuing drug treatment with isoniazid and rifampicin or isoniazid alone			
Monitoring treatment compliance with isoniazid and rifampicin or isoniazid alone			
Monitoring side effects of isoniazid and rifampicin or isoniazid alone			
Adjusting doses of isoniazid and rifampicin or isoniazid alone			

C. Latent TB: Enablers and barriers for management within primary care

13. There is a need for primary care-based, GP-led, latent TB treatment for healthy adult migrants:

    Strongly disagree / Disagree / Impartial / Agree / Strongly agree

14. Please indicate which of the following are potential barriers for you delivering latent TB treatment for healthy adult migrants in your primary care practice (*tick all those that apply*):

    ☐ Insufficient experience in managing patients with latent TB among GPs in your practice
    ☐ Lack of phlebotomy services in primary care
    ☐ Insufficient experience in ruling out active TB in migrants with positive mantoux of IGRA test
    ☐ Adult migrants are a difficult to engage patient group
    ☐ Long waiting times for GP appointments in the practice
    ☐ Insufficient experience in dealing with patients who are not taking their medication as directed
    ☐ Lack of experience interpreting blood tests for patients on latent TB treatment
    ☐ Lack of experience monitoring adverse drug reactions in patients on latent TB treatment
    ☐ Lack of experience managing treatment side effects in patients on latent TB treatment
    ☐ Lack of timely support from TB specialists/services
    ☐ Lack of available support from TB specialists/services
    ☐ Other, please specify _

    _

    _

Comments: _

_ _

_ _

_ _

15. Which enablers do you think would increase your willingness to deliver latent TB treatment for healthy adult migrants in your primary care practice (*tick all those that apply*):

    ☐ Tools to aid patient communications around risk
    ☐ Tools to aid patient communications around treatment side effects
    ☐ Tools to aid patient communications around the importance of treatment compliance
    ☐ Training - please specify: e-learning / workshops / handbook / other_ _ _ _ _ _ _ _ _ _ _ _ _ _ _ _ _ _ _
    ☐ More staff, please specify_ _

☐ More resources, please specify_ _

☐ Specific financial remuneration, please specify approximate amount _ _ _ _ _ _ _ _ _ _ _ _ _ _ _ _ _ _

☐ Easy access to support and advice services from local TB specialist teams

☐ Secondary care/TB specialist team oversight of management

☐ Having a primary care, nurse led latent TB screening service

☐ Other, please specify_ _

_ _

_ _

Comments: _

_ _

_ _

_ _

16. Which statement most accurately reflects your current willingness to deliver latent TB management for healthy adult migrants in primary care:

☐ I would be willing to manage latent TB patients in primary care under current circumstances

☐ I would potentially be willing to manage latent TB patients in primary care if my answers to Qu. 16 and 17 were considered in developing the service

☐ I would not be willing to manage latent TB patients in primary care under any circumstances

Any other comments: _

_ _

_ _

_ _

_ _

Thank you for completing this questionnaire.
